# Differences in the determinants of health insurance enrolment among working-age adults in two regions in Ghana

**DOI:** 10.1186/s12913-018-3192-9

**Published:** 2018-05-29

**Authors:** Stephen Kwasi Opoku Duku

**Affiliations:** 1 Department of Epidemiology, Noguchi Memorial Institute for Medical Research, University of Ghana, P. O. Box LG 581 Legon, Accra, Ghana; 2 Amsterdam Institute for Global health and Development, Amsterdam, The Netherlands; 3 Vrije Universiteit Amsterdam, Amsterdam, The Netherlands

**Keywords:** Health insurance, Determinants of enrolment, Rural-urban, National Health Insurance Scheme, Ghana

## Abstract

**Background:**

Ghana’s National Health Insurance Scheme (NHIS) has achieved varying levels of enrolment within the regions with different rural-urban populations with associated income inequalities. This study sought to investigate the differences in the determinants of enrolment between the Greater Accra (GAR) and Western (WR) regions of Ghana to inform the NHIS reforms.

**Method:**

Data from 4214 adults, 18 years and above from a household survey conducted in the two regions was analyzed. Bivariate analysis (*t*-test for continuous and Pearson chi-square for categorical) was performed to examine differences in respondents characteristics (socio-economic and insurance enrolment) between the two regions for the total, urban and rural samples. Logistic regression estimation was performed to establish differences in determinant of enrolment between the regions.

**Results:**

Age, sex, educational level, marital status, health status and travel time to nearest health facility were identified as determinants of enrolment in both regions and among the rural and urban residents within the regions. Although the rich and richest in both regions are more likely to enroll than the poor and poorest, the odds of enrolment for the urban richest in the WR is about twice that of GAR whiles the odds of enrolment for the rural richest in the GAR is also about twice that of the WR. Those who visit public facilities in the GAR are more likely to enroll than those in WR for the total and urban samples. However, those who visit private facilities in rural communities in both regions are more likely to enroll.

**Conclusion:**

Differences in the NHIS enrolment between the regions is as a result of differences in socio-economic factors that are intrinsic in the regions and impact on the inhabitants’ ability to afford insurance premium. Policymakers should determine NHIS premium differently at the district level based on socio-economic activities and income levels within the districts.

## Background

Health insurance has been recognized globally as one of the principal methods of financing healthcare to achieve universal coverage, particularly in low and middle income countries. Many low and middle income countries are currently exploring mechanisms of extending their health insurance schemes to specific groups to eventually cover their entire populations [1, 2]. The 2005 World Health Assembly resolution WHA 58.33 urged members states to ensure financial protection to all citizens, especially children and women of reproductive age and “to plan the transition to universal coverage of their citizens” [2]. Given the high demand for healthcare services of appreciable quality and the extreme under-utilization of health services in several Sub-Saharan African countries due to financial barriers, health insurance has been recommended as a promising alternative to other criticized financing systems like cost-recovery and user fees [2]. The expectation is that health insurance will improve access to quality healthcare through risk pooling of unforeseeable healthcare cost to fixed premiums [3]. In response to this call for developing countries to adopt healthcare financing mechanisms that remove financial accessibility barriers and strive towards the attainment of universal health coverage, Ghana implemented the National Health Insurance Scheme (NHIS) in 2004 to replace the “Cash and Carry” system when patients have to pay out-of-pocket cash before receiving healthcare.

The NHIS was established through an Act of parliament, Act 650 in 2003 as part of efforts to make the health goal within the Ghana Poverty Reduction Strategy (GPRS) achievable and also to accomplish the targets set in the Health Sector Five-Year Programme of Work, 2002–2006 [4–6]. The vision of the NHIS was to ensure equitable access to acceptable quality package of essential healthcare to all residents of Ghana [4]. Act 650 was revised in 2012 and replaced with Act 852 to remove administrative bottlenecks, introduce transparency, and reduce opportunities for corruption and gaming of the NHIS system [7]. The scheme is financed mainly from the National Health Insurance Fund (NHIF). Cash inflow into the NHIF is from 2.5% of the 17.5% Value Added Tax (VAT), 2.5% of the 17.5% Social Security and National Insurance (SSNIT) contributions made by formal sector employees, member contributions from premium payments and monies that accrue to the fund from investments made by the NHIA Council. The Government of Ghana also allocated funds to the NHIF through parliament and other donor funds [4–6]. Membership of the NHIS involves payment of registration fee and insurance premium before an NHIS Identity Card is issued. Formal sector employees are exempted from payment of premium and therefore have to pay only the registration fee to enroll. Informal sector workers have to pay the annual premium and registration fee (except those belonging to any of the exemption categories). Enrolment and financial contribution to the NHIS is legally mandatory by Act 852, but in practice it is voluntary as there are no penalties for non-enrolment [7]. What actually pertains is that the mandatory enrolment and contribution only applies to formal sector employees who pay Social Security and National Insurance Trust (SSNIT) contributions. Informal sector workers who form the majority of the Ghanaian population have to voluntarily pay the registration fees and premium to enroll in the NHIS.

After more than a decade of implementation, the NHIS has made significant progress in extending health insurance to the people of Ghana. The active membership of the scheme increased from 1.3 million in 2005 to 10.15 million in 2013, representing 38% of the Ghanaian population [8]. There are however, wide variations in enrolment coverage between the 10 regions with different rural and urban populations [8, 9]. The implementation of the NHIS also saw a steady increase in outpatient utilization of healthcare services. Outpatients’ utilization increased from under 5 million in 2005 to approximately 24 million in 2012 [9].

Ghana is considered as a rural country with approximately (49%) of the population living in rural areas with limited socio-economic opportunities [10]. Significant inequalities persist between the rural and urban areas in terms of availability of basic amenities and infrastructure such as water, sanitation and health facilities [10, 11]. In 2009, only about 10% of the urban population in Ghana lack access to portable water as compared to 26% of the rural population. Whilst 18% of urban population had access to improved sanitation, only 7% of the rural population had improved sanitation [11]. Large income inequalities also exist between rural and urban populations in Ghana. The overall poverty rate per capita is 39% for rural areas and 10% for urban areas. The rural areas also have severe poverty rate per capita of 25% as compared to 5% in urban areas [11]. Low productivity and poorly functioning markets for agricultural products have been cited as the main reason for the poverty gap between urban and rural Ghana [11]. The NHIS was therefore designed as a pro-poor initiative to ensure financial protection of the vulnerable in society including women and children in rural areas, with a graduation premium based on socio-economic status. However, in reality, premiums are generally flat rated at the district levels due to the general difficulty in classifying subscribers according to their relative socio-economic status [12].

Several studies have examined the determinants of health insurance enrolment and identified economic factors, socio-demographic factors, place of residence, behavioral factors and household size as some of the important determinants. Income, employment, and wealth index are very important economic determinants of health insurance enrolment. Cameron et al. [13], Asenso et al. [14], Sanhueza and Ruiz-Tagle [15] and Ying et al. [16], examined the relationship between income and health insurance. They all concluded that income predicts health insurance purchase. Economic theory shows that income has a fundamental influence on the decision to purchase health insurance as a “normal good”. Thus higher income increases the affordability of health insurance premium because at higher income, the opportunity cost of insurance purchase reduces [17, 18]. Other studies found that education and employment have direct positive correlation with health insurance enrolment [19–21].

Socio-demographic determinants of enrolment such as age, gender, education and marital status are often used to explain why some individuals may be employed, have high income and in high wealth index but still does not to enroll in health insurance. Economic theory indicates that as individuals’ age, they experience depreciation in their health stock and tend to purchase health insurance as an investment in health to avoid catastrophic health expenditure in the event of ill health [22]. However, the empirical evidence on the effect of age on health insurance enrolment has presented inconsistent results. While Mwaura [23], Bhat and Jain [24], Kronick and Gilmer [25], Savage and Wright [26] and Ayitey et al. [27] found advanced age to increase the likelihood of health insurance enrolment, Ying et al. [16], Jutting [25] and Brugiavini and Pace [28] found that being young increases the probability of health insurance enrolment. Other studies also found that higher level of education and being married also increases the likelihood of health insurance enrolment [14, 16, 23, 27–29]. A study by Muurinen [30] however reported a contrary finding that the highly educated are less likely to purchase health insurance and explained that highly educated people are likely to be healthier with low probability of risk, hence will have lower likelihood of health insurance enrolment. Studies by Butler [31] and Ayitey [14] revealed that people who are employed and those on executive positions have a higher probability of health insurance enrolment.

The evidence on the effect of gender on health insurance enrolment have reported inconsistent findings. While Asenso et al. [14] and Bourne & Kerr-Campbell [29] found that male headed households and being male increases the likelihood of health insurance enrolment, Ayitey et al. [27], Jutting [32] and Mwaura [23] found that female headed households and females are more likely to enroll in health insurance. The literature is however emphatic that married couples are more likely to enroll in health insurance [27, 33]. Similarly, larger household sizes have been reported to significantly increase the probability of health insurance enrolment [27, 34, 35]. Religion has also been reported as a significant predictor of health insurance enrolment [36].

Again the empirical evidence on the effect of residential locality on health insurance enrolments has been inconsistent. While some studies found little or no difference in rural-urban health insurance enrolment [36, 37], others found residential remoteness (rural areas) to be a significant determinant of health insurance enrolment [38–44]. Yet, other studies also found urban locality of residence to be a significant determinant of health insurance enrolment [14, 17, 45–49]. In terms of effect of health status and frequency of health facility visits on health insurance enrolment, the empirical evidence shows that individuals who are ill are more likely to enroll in health insurance and so are those who make more health facility visits [1, 27, 50].

From the evidence available in the literature, it can be deduced that the demographic characteristics of the inhabitants and the socio-economic differences that exist within the rural and urban areas and between the different regions of Ghana may be responsible for the observed differences in health insurance enrolment in the regions. Although several studies have been conducted to identify the determinants of health insurance enrolment in Ghana [12, 14, 20, 21, 27, 28, 34, 36, 44], to the best of my knowledge, no study has specifically examined the differences in these determinants between rural and urban communities and between the different regions of the country. This paper seeks to contribute to the broader understanding of determinants of health insurance enrolment in resource constraint setting. The paper compares the determinants of enrolment in two regions that are geographically similar but socio-economically different. The paper specifically assesses the differences in determinants of NHIS enrolment between the two regions for the total samples, the urban sample and finally the rural sample. This study will be relevant to policy makers as it will deepen their understanding of the factors that influence the decision to enroll in the NHIS differently in the different regions. This knowledge is expected to help policy makers decide on the best strategies to adopt to increase health insurance enrolment in the current era of NHIS reforms.

## Methods

### Study settings

This study uses data from the Client-oriented Health Insurance System in Ghana (COHEiSION) Project baseline survey that was conducted in the Greater Accra (GAR) and Western (WR) regions of Ghana in April 2012. These two coastal regions have similarities and differences as far as rural and urban populations and socio-economic activities are concerned. Table 1 presents the differences and similarities between the Greater Accra and Western regions. These two regions were purposively selected to provide rural/urban as well as socio-economic differences that are of interest to the study and allow the assessment of its impact on health insurance enrolment.

**Table 1:** Geographical and Socio-economic Comparison of Study Regions

Description	Greater Accra Region	Western Region
Topography	Coastal region	Coastal region
Total Population	4,010,054	2,376,021
Percentage rural Population	9.5% live in rural areas	57.6% live in rural areas
Percentage urban population	90.5% live in urban areas	42.4% live in urban areas
Jobs of inhabitants in Urban Areas	Mainly engaged in white collar jobs (Government Ministries, Departments and Agencies; manufacturing and large scale businesses)	Few are engaged in white collar jobs. Mainly engaged in small scale businesses (retailing)
Economic activities in rural areas	Predominantly fishermen, farmers, small scale salt producers and some small scale miners	Predominantly fishermen, farmers, small scale salt producers and some small scale miners
Regional poverty incidence	12%	18%
NHIS coverage	25.6%	32.2%

Source: 2011 NHIA Annual Report [9]; 2010 Population and Housing Census [10] and Gender Inequalities in Rural Employment in Ghana: An Overview [11]

### Data source

This study uses primary data from the baseline survey of COHEiSION project. Data was collected from 1920 randomly selected households within 10 km radius of selected primary healthcare facilities in the two regions. Respondents were sampled through a multistage sampling strategy. First 16 (8 in each region) districts with the same or almost same characteristics such as total Population, NHIS enrolment coverage, total number of accredited health centres/clinics and urban or rural categorization were selected for the project. Sixty-four (32 in each region) clusters of NHIS accredited primary healthcare facilities were then selected from the selected districts on the basis of their ownership (public/private), location (rural/urban) and NHIS accreditation quality scores. Subsequently, 30 households were randomly sampled from within a 10 km radius of each selected primary healthcare facility. This sampling process ensured that a selected facility is the only primary healthcare facility within the 10 km radius catchment area. It also satisfied the randomized controlled trial design of the COHEiSION project that required equal number of intervention and control health facilities. Respondents were contacted for the interview in April 2012. A semi-structured questionnaire was used to collect information on respondents’ socio-demographics, social capital and social schemas, employment status, health status and healthcare utilization behavior, NHIS enrolment status, consumption expenditure patterns and dwelling characteristics. In total, data on 7097 household members was generated from the survey. This paper analysis data on 4214 individuals who were 18 years and above.

### Theoretical framework

The Expected Utility Theory of demand for Insurance under conditions of uncertainty and risk aversion by Von Neuman and Morgenstern contends that health insurance enrolment decision is one of a discrete choice to enroll or not [51]. The theory assumes that individuals are risk averse and make choices between taking risk with different implications on wealth. Thus at the time of health insurance enrolment decision, individuals are uncertain about whether they will be ill or not and also of the financial implications should they become ill. Individuals enroll in health insurance to protect themselves from catastrophic health expenditures in the event of ill health. The expectation is that in the event of ill health, the cost of treatment will be covered by the health insurance and in most instances, this cost is more than the health insurance premium paid, representing a gain. The decision to enroll is therefore arrived at by comparing the expected utility with health insurance to expected utility without health insurance. Risk averse individuals prefer to pay a certain known amount as health insurance premium to uncertain amounts of the same expected utility in the event of ill health [52–54]. According to the expected utility theory, the demand for health insurance by risk averse individuals to avoid the risk of wealth loss should be higher than risk neutral individuals who are indifferent about health insurance enrolment and risk loving individuals who would not want to purchase health insurance [55, 56]. This paper will rely on the expected utility theory to understand the determinants of enrolment in the NHIS in Ghana.

### Empirical model

The focus of this study is to interpret the dependent variable as a likelihood of enrolling in health insurance or not given other explanatory variables. The logit model is employed in the empirical estimation. This is because the logit model is able to overcome the problems associated with the Linear Probability Model (LPM) which allow the use of Ordinary Least Square (OLS) to estimate the parameters. The LPM is plagued with heteroscedasticity, non-normality of the disturbance term, low R^2^ and non-fulfilment of the 0 ≤ (Y_i_ / X_i_) ≤ 1 restriction of binary models. The logit model has the advantages of being more robust such that the independent variables don’t have to be normally distributed or have equal variance in each group, does not assume a linear relationship between the independent and dependent variables, does not assume homogeneity of variance and does not assume normality of error term. The maximum likelihood method is used to estimate the parameters [27]. The functional model for the determinants of health insurance enrolment can be formulated as follows:

1 $$ {H}^{\ast }=f\left(D,L,{\uppi}_2,I\right) $$

Where,

*H*^∗^ = NHIS status of the individual.

D = Demographic characteristics of the individual.

L = Health status of the individual.

*π* = Probability of illness.

I = Individual’s income.

When the logit model is applied to eq. (1), it can be expressed as:

2 $$ Logit\ \left({H}^{\ast}\right)={\beta}_0+{\sum}_{i,j=1}^k{\beta}_j{X}_i+{\varepsilon}_{ij} $$

The assumption with the logit mode is that there is a continuous latent variable y* that determines enrolment in the NHIS. Thus if y* is positive, then the individual will enrol in the NHIS and the observed binary outcome is one (1), otherwise the outcome is zero (0). The latent variable y* is therefore modelled by a linear regression function of the individual [27]. The estimable equation is therefore formulated as:

$$ {\displaystyle \begin{array}{l} NHISstatus={\beta}_0+{\beta}_1 female+{\beta}_2{age}_i+{\beta}_3 nevermarried+{\beta}_4 otherreligion+{\beta}_5 Hhsize\\ {}+{\beta}_6 noformaledu+{\beta}_7 unemployed+{\beta}_8{poorhealthstatus}_i+{\beta}_9 poorestwealth\\ {}+{\beta}_{10} disttofacility+{\beta}_{11} public\end{array}} $$

### Measurement of variables

Current enrolment in the NHIS is defined as the dependent variable of interest for this study. It assumes a value of 1 if the individual is currently enrolled in the scheme otherwise 0. The Ghana Statistical Service (GSS), 2010 Population and Housing Census classification of rural and urban areas was used to classify individuals as living in either rural or urban area [4]. According to this classification, five (Ablekuma, Ayawaso, Tema, Kpeshie and Okaikoi) out of the eight selected districts in the Greater Accra Region are urban districts and the remaining three (Dangme East, Dangme West and Ga West) are rural districts. It also classified five (Bia, Amenfi East, Wassa West, Jomoro, and Ahantaman) out of the eight selected districts in the Western Region as rural and three (Amanfiman, Sekondi and Takoradi) as urban districts.

Other demographic, socio-economic, health status characteristics of respondents and characteristics of the health facilities that empirical evidence suggest can influence health insurance enrolment were included in the estimation as explanatory variables. The explanatory variables included in the estimation are age, gender, religion, marital status, household size, educational level, employment status, wealth status (a 5 quintile proxy measure for annual food and non-food household consumption expenditure per capita), health status, private/public health facility and time taken to move from home to the nearest health facility. Table 2 summarizes the dependent and independent variables used in the analysis and how they were measured.

**Table 2:** Measurement of Variables

Dependent Variable	Operational Measurement
Health insurance enrolment NHIS enrolment	0 = Uninsured and 1 = Insured 0 = currently uninsured and 1 = currently insured
Key Independent Variable	Operational Measurement
Rural-urban location	0 = Urban and 1 = Rural
Type of health insurance enrollment	0 = Other insurance and 1 = Enrolled in NHIS
Age	Continuous positive whole numbers in years
Sex	0 = Female and 1 = Male
Marital Status	1 = Never married, 2 = Married and 3 = Divorced
Religion	0 = Other religion and 1 = Christian
Household size	Continuous positive whole numbers of the number of household members
Educational Level	1 = No education, 2 = Basic education and 3 = Secondary education and above
Employment Status	0 = Unemployed and 1 = Employed
Type of Employment	0 = part-time employment and 1 = Full-time employment
Occupation	1 = Farmer, 2 = Artisan/Trader, 3 = Labourer/Casual, Managerial/Professional, 5 = Business Owner
Annual Income	Amount earned measured in Ghana Cedis
Wealth Status	1 = poorest; 2 = poor; 3 = average; 4 = rich and 5 = richest (per capita household food and non-food consumption expenditure)
Frequency of health facility visits	Continuous positive whole numbers of the number of health facility visits within the last 6 months prior to the survey
Health Status	1 = Bad health, 2 = Fair health and 3 = Good Health
Time taken from home to facility	Continuous positive whole numbers of the time in minutes it takes to move by road from home to health facility
Ownership of Health Facility	0 = Private and 1 = Public

### Data analysis

In analyzing the data, 4214 individuals for the age cohort of 18 years and above was used. First descriptive statistics was used to present respondents proportion/average demographic and socio-economic characteristics in the two regions for the total, urban and rural samples. Bivariate analysis (*t*-test for continuous and Pearson chi-square for categorical) was performed to examine differences in respondents characteristics (socio-economic and insurance enrolment) between the two regions for the total, urban and rural samples. Finally, logistic regression estimation was performed to identify the determinants of enrolment in the two regions for the total, urban and rural sample. Respondents who were enrolled in insurance schemes other than the NHIS were excluded from the regression estimation.

## Results

### Characteristics of respondents

Table 3 present respondents’ demographic, socio-economic and health status characteristics by rural and urban areas in the total, GAR and WR samples. The average age in the total sample is 38 years. The average age in the GAR is slightly higher (39 years) than their WR (37 years) counterparts. Urban adults in the GAR are older (39 years) than urban adults in the WR (37 years). Similarly, rural adults in the GAR are older (38 years) than rural adults in the WR. There are more females (56%) in the total sample than males. Similarly, there are more females in the GAR (57%) and WR (56%) respectively than males.

**Table 3:** Characteristics of Working-Age Adults by Rural and Urban Area in the Two Regions

		Total Sample (*N* = 4214)			Greater Accra Region (*N* = 2187)			Western Region (*N* = 2027)		
		Mean (N = 4214)	Urban (*N* = 2171)	Rural (*N* = 2043)	Mean (N = 2187)	Urban (*N* = 1407)	Rural (*N* = 780)	Mean (N = 2027)	Urban (*N* = 764)	Rural (*N* = 1263)
Mean Age		37.74	38.61	36.81	38.61	39.26	37.98	36.59	37.43	36.09
Sex (%)	Female	56.29	56.06	56.53	56.65	56.15	57.56	55.90	55.89	55.90
	Male	43.71	43.94	43.47	43.35	43.85	42.44	44.10	44.11	44.10
Marital Status (%)	Never Married	34.77	36.99	32.40	37.27	38.59	34.87	32.07	34.03	30.88
	Married	51.50	48.60	54.58	48.98	46.91	52.56	54.27	51.70	55.82
	Divorced	13.74	14.42	13.02	13.81	14.50	12.56	13.67	14.27	13.30
Religion (%)	Other Religion	10.39	9.44	11.40	10.52	10.31	10.90	10.26	7.85	11.72
	Christian	89.61	90.56	88.60	89.48	89.69	89.10	89.74	92.15	88.28
Mean Household Size		4.5	4.4	4.6	4.4	4.3	4.5	4.6	4.4	4.7
Educational Level (%)	No Education	13.69	9.95	17.67	10.75	7.18	17.18	16.87	15.05	17.97
	Basic Education	52.06	47.31	57.12	50.80	45.70	60.00	53.43	50.26	55.34
	Secondary Educ. and above	34.24	42.75	25.21	38.45	47.12	22.82	29.70	34.69	26.68
Employment (%)	Not Employed	30.45	33.12	27.61	32.14	34.47	27.95	28.61	30.63	27.40
	Employed	69.55	66.88	72.39	67.86	65.53	72.05	71.39	69.37	72.26
Type of Employment (%) (*N* = 2931)	Part-time	1.71	2.13	1.28	2.09	2.60	1.25	1.31	1.32	1.31
	Full-Time	98.29	97.87	98.72	97.91	97.40	98.75	98.69	98.68	98.69
Occupation (%) (*N* = 1931)	Farmer	18.85	10.22	27.34	8.41	3.44	16.76	29.40	22.01	33.63
	Artisan/Trader	46.85	49.05	44.69	51.95	51.94	51.96	41.70	44.02	40.38
	Laborer/Casual Worker	7.83	8.67	7.01	8.07	8.10	8.01	7.59	9.65	6.42
	Managerial/Professional	18.46	22.41	14.57	20.79	24.20	15.08	16.10	19.35	14.27
	Business Owner	8.01	9.68	6.28	10.78	12.32	8.19	5.20	5.02	5.31
Wealth Status (%)	Poorest	17.56	11.56	23.94	12.53	8.53	1974	22.99	17.15	26.52
	Poor	17.13	15.02	19.38	15.96	14.14	19.23	18.40	16.62	19.48
	Average	19.06	19.81	18.26	18.98	18.91	19.10	19.14	21.47	17.74
	Rich	22.19	24.74	19.48	22.95	23.95	21.15	21.36	26.18	18.45
	Richest	24.06	28.88	18.94	29.58	34.47	20.77	18.11	18.59	17.81
Average Annual Income		2340.76	2470.33	2203.07	2470.33	2256.41	2413.73	2371.23	2864.29	2072.97
Health Status (%)	Bad Health	4.77	4.65	4.89	5.21	5.90	3.97	4.29	2.36	5.46
	Fair Health	8.66	8.01	9.35	9.33	9.10	9.74	7.94	6.02	9.11
	Good Health	86.57	87.33	85.76	85.46	85.00	86.28	87.77	91.62	85.43
Ownership of Facility (%)	Private	39.44	20.73	59.32	38.45	18.62	74.23	40.50	24.61	50.12
	Public	60.56	79.27	40.68	61.55	81.38	25.77	59.50	75.39	49.88
Time to Health Facility		12.43	12.64	12.19	12.64	10.46	12.63	13.75	16.68	11.92

Source: COHEiSION Project Baseline Survey of 2012

*GAR* Greater Accra Region, *WR* Western region

There are more married respondents (51%) in the total sample, the GAR (49%) and WR (54%) respectively than the other categories of marital status. There are however, more married couples in both urban (47%) and rural (53%) GAR than in urban and rural WR. Majority (90%) of respondents in the total sample, GAR (89%) and WR (90%) are Christians. There are however slightly more Christians in urban WR (92%) than urban GAR (90%) whiles rural GAR (89%) has more Christians than rural WR (88%).

There are approximately 5 members per household in the total sample, 4 members in GAR and 5 members in WR respectively. Urban GAR and urban WR have the same household size of 4 members. Similarly, the average household size in rural GAR (5) is equal to average household size in rural WR (5). More than half of respondents in the total sample (52%), GAR (51%) and WR (53%) have completed basic level of education. The proportion of adults with basic level of education in urban GAR (46%) is less than their urban WR (50%) counterparts. Majority of respondents in the total sample (70%), GAR (68%) and WR (71%) are gainfully employed. Those employed in urban WR (69%) are more than those in urban GAR (66%). However, both rural GAR and rural WR have equal proportion of 72% employed adults. Almost all (more than 98%) of the employed adults in the total sample, GAR and WR are in full-time employment. Of those employed, 47% in total, 52% in GAR and 42% in WR samples are artisans/traders. There are more traders/artisans in urban GAR (52%) than urban WR (44%). Similarly, there are more artisans/traders in rural GAR (52%) than rural WR (40%).

The richest wealth quintile had the highest proportion of 24% of respondents in the total sample and 30% in GAR than other wealth quintile. However, in the WR, the highest proportion of 23% of respondents are in the poorest wealth quintile. The proportion of respondents in the poorest wealth quintile in urban GAR (8%) is far smaller than their urban WR (17%) counterparts. Conversely, the proportion of respondents in the richest wealth quintile in rural GAR (34%) is much higher than their rural WR (18%) counterparts. Majority of respondents in the total sample (87%), GAR (85%) and WR (88%) indicated that they are of good health. The average annual income from all sources for respondents in the total sample is GH₵2340.76, GAR (GH₵2470.33) and WR (GH₵2371.23). Whilst average annual income level in urban WR (GH₵2864.29) is higher than urban GAR (GH₵2256.41), average annual income in rural GAR (GH₵2413.73) is higher than their rural WR (GH₵2072.97) counterparts. The proportion of urban respondents in WR (92%) with good health status is more than their urban GAR (85%) counterparts. Conversely, the proportion of respondents in rural GAR (86%) with good health status is more than their rural WR (85%) counterparts. Most (61%) of health facilities in the total sample, GAR (62%) and WR (60%) were public owned facilities. Whiles urban GAR has more private (74%) than public (26%) health facilities, urban WR has approximately equal (50%) private and public health facilities. On the average, it takes approximately 12 min to move from home to the nearest health facility in the total sample, 13 min in GAR and 14 min in the WR. It takes less time to get to the health facility in urban GAR (10 min) than urban WR (17 min). However, it takes more time to get to the health facility in rural GAR (13 min) than in rural WR (12 min).

### Regional differences in characteristics by rural and urban samples

Table 4 presents the bivariate analysis of health insurance enrolment and other explanatory variables between the two regions for the total, urban and rural samples. The WR have a higher (54%) health insurance coverage than the GAR (45%) in the total sample. Health insurance coverage in the urban sample is higher for GAR (59%) than the WR (41%).

**Table 4:** Bivariate Analysis of Health Insurance, Independent Variables by Regional and Rural/Urban

		Total Sample (N = 4214)			Urban (N = 2171)			Rural (N = 2043)		
		GAR (N = 2171)	WR (N = 2043)	*p*-value	GAR (N = 1407)	WR (N = 764)	P-value	GAR (N = 780)	WR (N = 1263)	*P*-value
Enrolment in Health Insurance										
Uninsured (%)		56.51	43.49	0.000***	68.92	31.08	0.004**	43.04	56.96	0.019*
Insured (%)		45.35	54.65		58.82	41.18		31.47	68.53	
Type of Health Insurance										
Other Health Insurance (%)		60.18	39.82	0.032*	81.03	18.97	0.024*	38.18	61.82	0.429
NHIS (%)		44.32	55.68		57.26	42,74		31.01	68.99	
Average Age		38.79	36.59	0.002**	39.26	37.43	0.078	27.97	36.09	0.051*
Sex										
Females (%)		52.23	47.77	0.601	64.91	35.09	0.892	38.87	61.13	0.446
Males (%)		51.47	48.53		64.68	35.32		37.27	62.73	
Marital Status										
Never Married (%)		55.63	44.37	0.034*	67.62	32.38	0.319	41.09	58.91	0.366
Married (%)		49.31	50.69		62.56	37.44		36.77	63.23	
Divorced (%)		52.16	47.83		65.18	34.82		36.84	63.16	
Religion										
Other Religion (%)		52.51	47.49	0.889	70.73	27.27	0.260	36.48	63.52	0.786
Christian (%)		51.83	48.17		64.81	35.19		38.40	61.60	
Average Household Size		4.4	4.6	0.252	4.3	4.4	0.655	4.5	4.7	0.519
Educational Level										
No Education (%)		40.73	59.27	0.009**	46.76	53.24	0.006**	37.12	62.88	0.408
Basic Education (%)		50.64	49.36		62.61	37.39		40.10	59.90	
Secondary Educ. and above (%)		58.28	41.72		71.44	28.56		34.56	65.44	
Employment										
Unemployed (%)		54.79	45.21	0.122	67.45	32.55	0.263	38.65	61.35	0.857
Employed (%)		50.63	49.37		63.50	36.50		38.00	62.00	
Health Status										
Bad Health (%)		56.72	43.28	0.324	82.18	17.82	0.005**	31.00	69.00	0.493
Fair Health (%)		55.89	44.11		73.56	26.44		39.79	60.21	
Good Health (%)		51.23	48.77		63.08	36.92		38.41	61.59	
Wealth Status										
Poorest (%)		37.03	62.97	0.000***	47.81	52.19	0.005**	31.49	68.51	0.482
Poor (%)		48.34	51.66		61.04	38.96		37.88	62.12	
Average (%)		51.68	48.32		61.86	38.14		39.95	60.05	
Rich (%)		53.69	46.31		62.76	37.24		41.46	58.54	
Richest (%)		63.81	36.19		77.35	22.65		41.86	58.14	
Ownership of Facility										
Private (%)		50.60	49.4	0.869	58.22	41.78	0.696	47.77	52.23	0.204
Public (%)		52.74	47.26		66.53	33.47		24.19	75.81	
Average Time to Health Facility		11.23	13.75	0.106	10.46	16.68	0.018*	12.63	11.92	0.761

Source: COHEiSION Project Baseline Survey of 2012. For continuous variables, means are reported and significant test is based on t-test whiles for categorical variables percentages are reported and significant test is based on Chi-square test.; Note; * p < 0.05; ** *p* < 0.01; ***p < 0.001

However, in the rural sample, health insurance coverage is higher in the WR (69%) than the GAR (31%). These differences in insurance coverages are statistically significant at the 90% confidence interval. In terms of type of health insurance, the WR (56%) again have a higher NHIS coverage than the GAR (44%) in the total sample. For the urban sample, the GAR have a higher (57%) NHIS coverage than the WR (43%) whiles in the rural sample the WR have a significantly higher (69%) NHIS coverage than the GAR (31%).

The average age of respondents in the GAR (39 years) is significantly higher than the WR (37 years) in the total sample. There is however no statistically significant difference in the average age of respondents in the urban or rural samples. Similarly, there is no statistically significant difference in the proportion of females and males in either the total, urban or rural samples. Although in the total sample, there are more married respondents in the GAR (56%) than the WR (44%), the differences in the proportion of married respondents between the regions in the urban and rural samples are statistically insignificant as shown in Table 4. The proportion of respondents with basic level education is significantly higher in the GAR (51%) than the WR (49%) in the total sample. Similarly, in the urban sample, the proportion of respondents with basic level education is higher in the GAR (63%) than the WR (37%). There is however no statistically significantly difference in the proportion of respondents with basic level education between the GAR and WR in the rural sample. In terms of health status, there is no statistically significant difference in the categories of health status between the GAR and WR for the total and rural samples. The GAR however, have higher proportions the health status categories than the WR in the urban sample. The proportion of respondents in the richest wealth quintile is significantly higher in the GAR (64%) than the WR (36%) in the total sample. Similarly, the proportion of respondents in the richest wealth quintile is significantly higher in the GAR (77%) than the WR (23%). However, there is no statistically significant difference in wealth status categories between the GAR and WR in the rural sample.

There is also no statistically significant differences between the GAR and WR in terms of religion, employment, health facility ownership and average time to health facility either for the total, urban or rural samples.

### Determinant of health insurance enrolment

Table 5 presents the logistic regression estimations of the determinant of NHIS enrolment among working-age adults for the total, urban and rural samples. Estimating individuals’ income in developing countries is difficult and unreliable because most people are reluctant to disclose their true income. A five quintile wealth status was therefore computed from household food and non-food consumption expenditure as a proxy for income levels and used in the regression estimation. Again due to the inability of respondents to accurately determine the distance in kilometers from their home to the nearest primary healthcare facility, time taken in minutes to move from home to the nearest primary healthcare facility was used as a proxy to measure distance from home to health facility. The differences in the determinants of enrolment between the two regions are presented.

**Table 5:** Determinant of Health Insurance Enrolment

		Adjusted Odds Ratio					
		Total Sample		Urban Sample		Rural Sample	
Independent Variables		GAR	WR	GAR	WR	GAR	WR
Sex	Males	0.66*** (0.53–0.82)	0.52*** (0.44–0.63)	0.74** (0.57–0.97)	0.49*** (0.36–0.67)	0.46*** (0.34–0.64)	0.55*** (0.42–0.71)
Age		1.02*** (1.01–1.03)	1.03*** (1.02–1.04)	1.01** (1.01–1.03)	1.04*** (1.02–1.05)	1.01* (0.99–1.04)	1.02*** (1.01–1.04)
Marital Status	Married	1.28 (0.93–1.76)	1.14 (0.84–1.56)	1.14 (0.82–1.58)	0.86 (0.59–1.26)	1.60 (0.76–3.38)	1.29 (0.77–2.13)
	Divorced	1.11 (0.71–1.74)	0.66* (0.44–0.99)	0.96 (0.60–1.52)	0.42** (0.21–0.85)	1.22 (0.41–3.62)	0.73 (0.45–1.20)
Religion	Christian	1.08 (0.67–1.77)	1.10 (0.78–1.56)	1.20 (0.67–2.13)	1.08 (0.70–1.67)	0.68 (0.29–1.58)	1.05 (0.63–1.76)
Household Size		1.04 (0.98–1.10)	1.08 (0.98–1.18)	1.03 (0.94–1.13)	1.15* (0.99–1.31)	1.03 (0.93–1.16)	1.04 (0.92–1.18)
Educational Level	Basic Education	0.89 (0.63–1.28)	1.18 (0.89–1.57)	0.93 (0.50–1.72)	1.87* (1.06–3.31)	0.89 (0.59–1.34)	0.99 (0.75–1.32)
	Secondary Edu. & Above	1.49* (1.00–2.21)	1.61* (1.06–2.44)	1.37 (0.75–2.52)	1.95 (0.87–4.39)	2.07*** (1.33–3.22)	1.67* (1.05–2.66)
Employment	Employed	0.82 (0.61–1.11)	0.79 (0.61–1.03)	0.79 (0.55–1.13)	0.84 (0.52–1.39)	0.85 (0.49–1.47)	0.79 (0.55–1.14)
Health Status	Fair Health	0.84 (0.53–1.34)	0.63 (0.26–1.53)	0.85 (0.45–1.62)	0.08 (0.01–0.72)	1.10 (0.60–2.02)	0.87 (0.35–2.19)
	Good Health	0.60* (0.39–0.96)	0.54 (0.22–1.34)	0.58 (0.32–1.08)	0.06 (0.01–0.59)	0.82 (0.33–2.03)	0.80 (0.33–1.95)
Wealth Status	Poor	0.91 (0.49–1.67)	1.47 (0.88–2.46)	0.67 (0.29–1.54)	2.54* (0.99–6.52)	0.98 (0.38–2.55)	1.07 (0.57–2.03)
	Average	1.11 (0.60–2.04)	1.43 (0.90–2.26)	0.66 (0.28–1.58)	1.49 (0.56–3.97)	1.75 (0.75–4.13)	1.25 (0.72–2.17)
	Rich	1.35 (0.72–2.50)	1.77* (1.04–3.02)	1.16 (0.47–2.86)	2.68 (0.84–8.49)	1.04 (0.45–2.42)	1.26 (0.68–2.34)
	Richest	1.73* (0.97–3.10)	2.04** (1.26–3.28)	1.13 (0.49–2.63)	2.59 (0.93–7.22)	2.55** (1.16–5.59)	1.76* (1.04–2.98)
Travel time to Facility		1.00 (0.98–1.02)	0.99 (0.98–1.00)	1.00 (0.98–1.03)	0.99 (0.99–1.01	1.01 (0.98–1.04)	0.98** (0.97–0.99)
Ownership of Facility	Public	1.83*** (1.55–2.16)	0.93 (0.82–1.06)	1.78*** (1.39–2.26)	0.95 (0.82–1.11)	0.48*** (0.31–0.73)	0.31*** (0.26–0.37)
Constant		0.14*** (0.06–0.33)	0.34* (0.11–0.04)	0.17*** (0.05–0.59)	1.08 (0.14–8.65)	0.366 (0.09–1.58)	0.42 (0.11–1.59)
Pseudo R-squared		0.086	0.079	0.075	0.102	0.135	0.086
No. of Observation		2132	1946	1336	736	735	1167

Source: COHEiSION Project Baseline Survey of 2012. Note: Adjusted odds ratio are reported with reference categories: Age (1 year increase in age); Sex (Female); Marital status (Never Married); Religion (Other Religion); Household size (Increase of 1 person); Educational level (No Formal Education); Employment (Unemployed); Health Status (Poor Health Status); Wealth status (Poorest); Travel time (1 min increase in travel time); Facility ownership (Private); Missing values are excluded from the analyses. Note: * p < 0.05; ** *p* < 0.01; ***p < 0.001

The results show that generally, age, sex, marital status, educational level, health status, wealth status and health facility ownership are significant determinants of NHIS enrolment at varying extents between the GAR and the WR for the total, urban and rural samples. For the total sample, females are significantly more likely to enroll in the NHIS than males for both the GAR and WR.

Similarly, for both the urban and rural samples, females are significantly more likely to enroll in the NHIS than males in the GAR and WR. These findings are consistent with findings by Mwaura [23], Ayitey et al. [27] and Jutting et al. [32]. Age also impacts positively on NHIS enrolments. The results show that an increase of 1 year in age significantly increases the odds of NHIS enrolment in both regions for the total, rural and urban samples. These findings are consistent with findings by Bhat and Jain [24], Kronick and Gilmer [25], Savage and Wright [26] and Ayitey et al. [27]. In terms of marital status, for the total sample, whiles divorcees are 1.08 (CI = 0.67–1.77) times more likely to enroll in the NHIS in the GAR, their WR counterparts are 0.66 (CI = 0.44–0.99) times significantly less likely to enroll. For the urban sample, although divorcees in both regions are less likely to enroll as compared to those married, the results for the WR is statistically significant at the 95% confidence interval. However, for the rural sample, whiles divorcees in the GAR are 1.22 (CI = 0.41–3.38) more likely to enroll, their WR counterparts are 0.73 (CI = 0.45–1.20) less likely to enroll even though this result is statistically insignificant at the 90% confidence interval. This finding is in contrast with earlier studies by Asenso Okyere et al. [14], Ayitey et al. [27] and Brugiavini and Pace [28] who found married individuals to be significantly more likely to enroll in health insurance. Although the results further shows that being married increases the odds of NHIS enrolment in both regions for the total, urban and rural samples, these findings are statistically insignificant at the 90% confidence interval. Similarly, although the results show that being a Christian and a 1 member increase in household sizes increases the odds of NHIS enrolment, these findings are statistically insignificant even at the 90% confidence interval.

The results also show that an individuals’ level of education influence the odds of NHIS enrolment. For the total sample, having secondary level education and above significantly increases the likelihood of NHIS enrolment by 1.49 (CI = 1.00–1.11) times in the GAR and 1.62 (CI = 1.06–2.44) times in the WR respectively as compared to individuals with no formal education. For the urban sample, although the results shows that secondary level education and above increases the odds of enrolment by 1.37 (CI = 0.75–2.52) times in the GAR and 1.95 (CI = 0.87–4.39) times in the WR, these findings are statistically insignificant at the 90% confidence interval. However, for the rural sample, individuals with secondary level education and above are significantly more likely to enroll in the NHIS by 2.07 (CI = 1.33–3.22) times in the GAR and 1.67 (CI = 1.05–1.14) times in the WR. These findings are consistent with findings by Asenso Okyere et al. [14], Ayitey et al. [27] and Brugiavini and Pace [28]. The results show being employed does not positively influence NHIS enrolment in both regions for the total, urban or the rural samples. These findings are however statistically insignificant at the 90% confidence interval.

Although the finding was statistically insignificant, individuals with poor self-assessed health status are more likely to enroll in the NHIS than those with fair or good health status in both regions for the total, urban and rural samples. Ayitey et al. [27] reported similar findings of the effect of self-assessed health status on NHIS enrollment. The results show that wealth status is a significant determinant of NHIS enrolment at varying extent in the two regions depending on the rural/urban locality of residence of the individual. For the total sample, the richest in the GAR are 1.73 (CI = 0.97–3.10) times more likely to enroll in the NHIS than the poorest whiles in the WR the rich and the richest are 1.77 (CI = 1.04–3.02) times and 2.04 (CI = 1.26–3.28) times respectively more likely to enroll in the NHIS than the poorest. However, when the urban and rural samples are considered separately, whiles in the rural sample, individuals in the richest wealth quintile in the GAR are 2.55 (CI = 1.16–5.59) times and WR are 1.76 (CI = 1.04–2.98) times significantly more likely than those in the poorest quintile, in the urban sample individuals in the richest wealth quintile in both regions are more likely to enroll. These finding are statistically insignificant at the 90% confidence interval. These findings are in agreement with findings by Ayitey et al. [27] and Fowler et al. [50]. In terms of travel time from home to the nearest primary healthcare facility, although a 1 min increase in the travel time reduces the odds of NHIS enrolment of individuals in the WR for the total, urban and rural samples, these findings were statistically insignificant at the 90% confidence interval. From the results, ownership of the nearest primary healthcare facilities is another significant determinant of NHIS enrolment. Individuals who live around the catchment area of a publicly owned primary health facility in the GAR are 1.83 (CI = 1.55–2.16) times and 1.78 (CI = 1.39–2.26) times significantly more likely to enroll in the NHIS for the total and urban samples respectively. However, in the rural sample, individuals who live in the catchment area of private facilities are significantly more likely to enroll in the NHIS than those who live around public facilities.

## Discussion

The study assessed the differences in determinants of NHIS enrolment among working-aged adults in the GAR and WR in Ghana. The findings indicates that age, sex, educational level, marital status, health status, wealth status and health facility ownership are significant determinants of NHIS enrolment.

Females in both regions are more likely to enroll compared to males. Similarly, urban and rural females in both regions are more likely to enroll in the NHIS than males. This may be as a result of the Free Maternal Health Policy under the NHIS which offer premium exemption to expectant and nursing mothers and therefore most females in the reproductive age-group might have enrolled under this exemptions category. The likelihood of NHIS enrolment was also found to increase with age for both regions in the total sample and similarly for both regions in the urban and rural areas. This positive relationship of increasing age with NHIS enrolment can be attributed to degeneration in health as people age and the need for increased healthcare utilization. Older people therefore prefer to make investment in their health through the purchase of health insurance. This findings is consistent with the findings of previous studies [20, 27, 36]. The increased likelihood of the aged to health insurance enrolment can also be attributed to the exemptions of people aged 70 years and above from premium payment under the NHIS exemption policy. Most of the aged therefore enrolled under the 70+ age exemption category. Married people are more likely to enroll in the NHIS in both regions for the total sample and similarly in both regions in the urban and rural areas than never married people. This may be because married couples purchase health insurance to mitigate the financial burden that is likely to accrue from raising children after marriage [27, 31, 33].

The findings also point to educational class dimensions in NHIS enrolment. The better educated in both regions in the total and rural sample are significantly more likely to enroll than the uneducated. Although the results from the urban sample is statistically insignificance, there is still a positive relationship between higher education and NHIS enrolment in both regions. This positive relationship between higher education and NHIS is because higher education makes people better understand and appreciate the insurance concept and its benefits to the household. People with poor self-assessed health status in both regions are more likely to enroll in the NHIS in the total and urban samples than those with fair and good health status. However, in the rural sample, people who are of fair health status in the GAR are more likely to enroll in the NHIS than their WR counterparts. This suggest that people who are of poor health in urban communities on both regions self-select into the NHIS. Perhaps the availability of both public and private health facilities in urban communities is such that people of poor health get to know of their underlying propensity to increased healthcare utilization. They therefore enroll in the NHIS to protect themselves from catastrophic healthcare expenditure. Theoretical concepts such as adverse selection, risk aversion, affordability and trust which are beyond the scope of this study can help explain and put these findings into proper perspective.

Working-age adults in the rich and richest quintiles in both regions in the total sample are significantly more likely to enroll in the NHIS than those in the poorest wealth quintile. This suggest that wealth status and therefore affordability of insurance premium is an important determinant of NHIS enrolment. In the urban sample, the odds of NHIS enrolment for WR individuals in the rich and richest quintiles is about twice that of those in the GAR. Conversely, in the rural sample, the odds of NHIS enrolment for GAR individuals in the richest quintile are about twice that of those in the WR. Thus the rich in urban WR enroll more in the NHIS than the rich in urban areas in the GAR. However, in the rural areas, the rich in rural GAR enroll more in the NHIS than the rich in rural WR. The expectation is that with the pro-poor design of the NHIS and its extensive exemption policy that exempt premium payment for indigents, the poor in rural areas in both regions will enroll more than the rich. These finding further galvanize findings by earlier studies that poverty is a major barrier to NHIS enrolment and that the NHIS is not pro-poor as envisaged [27]. The fact that the odds of enrolment among the rural rich in the GAR is higher than rural rich in WR further indicates that levels economic activities, income levels and affordability of premium in the rural areas also impact on the decision to enroll in the NHIS. Although the WR has a high poverty rate of 18% compared to the GAR 12%, the NHIS enrolment rate in the WR (55.7%) is higher than the GAR (44.3%). However, the likelihood of enrolment is significantly higher among the richest in the WR (OR = 2.04) than in the GAR (OR = 1.73). This can be explained from the fact that the richest in the WR do not have access to many private health facility and private health insurance companies like the richest in the GAR. So even if the richest in WR are not satisfied with the quality of care from public health facilities which they will attend should they enroll in the NHIS, they will still have to attend these same public facilities should they opt out of the NHIS and pay out-of-pocket or enroll with other private health insurance. Unlike the GAR where the richest have access to many private insurance companies and private healthcare facilities, the richest who decide to opt out of the NHIS for quality issues have readily available alternatives.

Travel time from home to health facility is another important determinant of NHIS enrolment between the regions. The average travel time to the nearest health facility is higher in the WR than the GAR for the total, urban and rural samples. It is therefore not surprising that a 1 min increase in the travel time in the WR reduces the odds of NHIS enrolment for the total, urban and rural samples. The ownership of the nearest primary health facility is another important determinant of NHIS enrolment. People who visit public health facilities in the GAR are more likely to enroll in the NHIS than their WR counterparts for the total and urban samples. However in the rural areas, this is not the case as those who visit private facilities are significantly more likely to enroll in the NHIS in both regions. A possible explanation to this could be about perceived poor quality care from these public facilities which may influence peoples decision to enroll in the NHIS. This is because per the NHIS gate keeping system, card holders are expected to first visit a primary health facility when sick. If people perceive the quality of care they receive from their nearest primary health facility to be poor, chances are that it will discourage them from enrolling in the NHIS.

## Conclusion

The study assessed the differences in determinants of NHIS enrolment among working-aged adults in the GAR and WR in Ghana. The analysis employed logistic regression in the empirical estimation. The study did not find any differences in the demographic determinants of NHIS enrolment between the two regions and among the rural and urban residents in the two regions. The findings indicate that generally, age, sex, educational level, marital status, health status and travel time to health facility are significant determinants of NHIS enrolment in both regions and similarly in the rural and urban communities in the two regions. The study however found some differences between the two regions in terms of wealth status and health facility ownership as determinants of NHIS enrolment. Although the rich and richest in both regions are more likely to enroll in the NHIS than the poor and poorest, the odds of NHIS enrolment for the richest in urban areas in the WR (OR = 2.59) is about twice as that of GAR (OR = 1.13) whiles in the rural areas the odds of NHIS enrolment in the GAR (OR = 2.55) is also about twice that of the WR (OR = 1.76). People who visit public health facilities in the GAR are more likely to enroll in the NHIS than those in WR for the total and urban samples. However in the rural areas, those who visit private facilities are significantly more likely to enroll in the NHIS in both regions. These findings suggest that inequalities still exists in NHIS enrolment in favor of the wealthy, communities with better socio-economic activities and communities with health facilities that are perceived to provide better quality healthcare. This thus raises concerns as to whether the NHIS is truly pro-poor as envisaged.

The study contributes to the literature on the determinants of NHIS enrolment by identifying factors that might be responsible for the observed differences in the NHIS regional enrolment coverages. The differences in NHIS enrolment coverages between the regions may be as a result of differences in socio-economic factors that impact on the ability of the inhabitants to afford the insurance premium. This should serve as an important indicator to policymakers on the need to focus on regional specific geographic and proxy means targeting strategies aimed at identifying the poor in resource constraints communities for premium exemptions. The study recommends that policymakers should use innovative approaches to determine NHIS premium at the district level based on socio-economic activities and income levels within the district. They should also consider introducing quality healthcare dimension into provider payment mechanisms to rewards providers who meet quality criteria as expressed by their clients. This will serve as an incentive for both public and private accredited healthcare providers to provide quality healthcare that attract individuals to enroll in the NHIS.

Finally, the findings of this study is subject to the following limitations. The analysis relied on self-reported measures such as health insurance enrolment status, patterns of enrolment and self-assessed health status. Therefore any systematic differences due to respondents reporting bias could affect the precision of the reported estimates. The study did not include individuals insured with other private insurance companies other than the NHIS due to the small sample size in the data. These limitation however does not invalidate the entirety of the self-reported measures and the analysis of these measure which have been well-documented. The findings of the determinants of NHIS enrolment reported in this study are limited to the Greater Accra and Western regions of Ghana and therefore caution should be taken when generalizing the findings to other populations.

## Abbreviations

COHEiSION Project: Client-Oriented Health Insurance System in Ghana Project
GAR: Greater Accra Region
GSS: Ghana Statistical Service
NHIA: National Health Insurance Authority
NHIF: National Health Insurance Fund
NHIS: National Health Insurance Scheme
NOW: Netherlands Organization for Scientific Research
SSNIT: Social Security and National Insurance Trust
WHA: World Health Assembly
WOTRO: Science for Global Development
WR: Western Region
